# Dysregulation of Human β-Defensin-2 Protein in Inflammatory Bowel Disease

**DOI:** 10.1371/journal.pone.0006285

**Published:** 2009-07-20

**Authors:** Marian C. Aldhous, Colin L. Noble, Jack Satsangi

**Affiliations:** Gastrointestinal Unit, School of Clinical and Molecular Medicine, University of Edinburgh, Institute of Genetics and Molecular Medicine, Western General Hospital, Edinburgh, Scotland, United Kingdom; Charité-Universitätsmedizin Berlin, Germany

## Abstract

**Background:**

Human β-defensin-2 (HBD2) is an antimicrobial peptide implicated in the pathogenesis of inflammatory bowel disease (IBD). Low copy number and concomitant low mRNA expression of the HBD2 gene have been implicated in susceptibility to colonic Crohn's Disease (CD). We investigated the colonic distribution of HBD2 mRNA expression, and the contributions of genetic and environmental factors on HBD2 protein production.

**Methodology/Principal Findings:**

We examined HBD2 mRNA expression at three colonic locations by microarray analysis of biopsies from 151 patients (53 CD, 67 ulcerative colitis [UC], 31 controls). We investigated environmental and genetic influences on HBD2 protein production using *ex vivo* cultured sigmoid colon biopsies from 69 patients (22 CD, 26 UC, 21 controls) stimulated with lipopolysaccharide (LPS) and/or nicotine for 24 hours. HBD2 and cytokines were measured in culture supernatants. Using DNA samples from these patients, regions in the HBD2 gene promoter were sequenced for NF-κB binding-sites and HBD2 gene copy number was determined. HBD2 mRNA expression was highest in inflamed (vs. uninflamed p = 0.0122) ascending colon in CD and in inflamed (vs. uninflamed p<0.0001) sigmoid colon in UC. HBD2 protein production was increased in inflamed UC biopsies (p = 0.0078). There was no difference in HBD2 protein production from unstimulated biopsies of CD, UC and controls. LPS-induced HBD2 production was significantly increased in CD (p = 0.0375) but not UC (p = 0.2017); this LPS-induced response was augmented by nicotine in UC (p = 0.0308) but not CD (p = 0.6872). Nicotine alone did not affect HBD2 production. HBD2 production correlated with IL8 production in UC (p<0.001) and with IL10 in CD (p<0.05). Variations in the HBD2 promoter and HBD2 gene copy number did not affect HBD2 production.

**Significance/Conclusions:**

Colonic HBD2 was dysregulated at mRNA and protein level in IBD. Inflammatory status and stimulus but not germline variations influenced these changes.

## Introduction

The inflammatory bowel diseases (IBD), Crohn's disease (CD) and ulcerative colitis (UC) are common causes of chronic disease in the developed world and represent an important public health issue, with a combined prevalence in northern Europe estimated at 1 in 250 [1]. The incidence and prevalence of both adult and early onset disease is particularly high in Scotland [2] affecting quality of life and employment potential in adults as well as growth and education in children.

Considerable recent progress has been made in understanding the pathogenesis of IBD. A number of susceptibility genes have been identified [3], [4] highlighting important pathogenic mechanisms, notably the innate immune response in CD. Additionally, there is now unequivocal evidence regarding the identity of environmental influences involved in the pathogenesis of IBD, with consistent data implicating the gut flora, as selective changes in bacterial populations have been demonstrated in patients with different phenotypes of CD [5], [6].

Smoking is the best known environmental factor implicated in the pathogenesis of IBD [7]. We and others have illustrated that smoking habit strongly affects the disease history of both CD [8]–[10] and UC [11], [12]. Of the>4000 chemicals in cigarette smoke, nicotine has been studied as a constituent potentially affecting intestinal inflammation. Studies of transdermal nicotine therapy suggest that this may be effective in UC patients with active disease [13], [14]. However, the mechanisms whereby nicotine may influence intestinal inflammation are poorly characterised and there have been few studies of the effects of nicotine on the cellular responses of IBD patients, especially in CD. In mouse models of IBD, nicotine had differential effects on the small and large bowel [15], [16], with increased jejunal inflammation and decreased colonic inflammation together with inhibition of pro-inflammatory cytokines [17].

The interest in the innate immune response in disease pathogenesis has led to evaluation of the role of defensins, part of a family of anti-microbial peptides with direct bactericidal activity against a wide variety of bacteria [18]. In man, α-defensins, HD-5 and HD-6 are expressed in small-bowel Paneth cells [19]. Production of α-defensins is dysregulated in CD [20]–[22], and we have also demonstrated them to be up-regulated in UC due to Paneth cell metaplasia [23]. The human β-defensins (HBDs) are expressed in various tissues throughout the body, with HBD1, HBD2 and HBD3 expressed in the gut [24]. Studies at the mRNA level, have shown that HBD1 is normally constitutively expressed but reduced in CD and UC patients and that both HBD2 and HBD3 are induced with inflammation in CD and UC [25], [26]. Other studies have indicated that probiotic bacteria induce HBD2 production in intestinal epithelial cells via NF-κB, leading to increased barrier function in the gut [27], [28]. Taken together these data suggest that β-defensins, in particular HBD2, may have a key role in determining innate immune responses to bacteria in the gut.

Genetic factors have also been implicated in HBD2 protein production. Defensins, on chromosome 8p23, have been found to be polymorphic for copy number [29], [30]. Recent work has shown an association between high copy number of the HBD2 gene *DEFB4* and risk of psoriasis, suggesting that HBD2 may be associated with an inappropriate inflammatory response in the skin [31]. As HBD2 production has been shown to be abrogated by deletion of NF-κB binding sites in the *DEFB4* promoter region [28], the relative contribution of inflammation, perhaps via activation of NF-κB in IBD [32] is potentially important in the regulation of HBD2 production. In the only study to date of IBD patients, Fellerman *et al*., suggested that low copy number of the HBD2 gene, *DEFB4*, predisposed to colonic disease [33] and that copy number was associated with levels of mRNA expression in inflamed rectal biopsies. However, these data have not been independently replicated and the relationship between *DEFB4* copy number and HBD2 protein production has not been investigated in IBD.

In this study, we initially mined microarray expression data [23] previously obtained from our Scottish patient population and showed that expression of HBD2 mRNA changed with disease, inflammation and colonic location. We then investigated production of HBD2 protein from biopsies in *ex vivo* organ culture and assessed the effects of environmental factors, such as inflammation status and stimulus (bacterial lipopolysaccharide [LPS] and/or nicotine) and genetic factors (NF-κB binding sites and *DEFB4* copy number) that might affect this production. Our results show that inflammation and stimulation had more of an effect on HBD2 production than these genetic factors.

## Materials and Methods

### Ethics statement

The Medicine and Oncology Subcommittee of the Lothian Local Research Ethics Committee approved the study protocol (LREC 2001/4/72).

### Patients and controls

Full details of the patients involved in the microarray analysis are given elsewhere [23] (and Noble 2009, submitted to Gut). Data were used of biopsies taken from 3 colonic locations (ascending, descending or sigmoid colon) from patients with CD (83 biopsies from 53 patients), UC (124 biopsies from 67 patients) or controls (66 biopsies from 31 patients). Of the controls, 23 had histologically normal biopsies, while 8 had inflamed colonic biopsies as defined from a paired biopsy graded by an experienced gastrointestinal pathologist as having no evidence of inflammation, or with evidence of acute/chronic inflammation and/or acute/chronic inflammatory cell infiltrate.

For the *ex vivo* organ culture studies, IBD patients (22 CD, 26 UC) underwent routine colonoscopy indicated in their clinical management at the Western General Hospital, Edinburgh, Scotland. The diagnosis of IBD adhered to Lennard-Jones criteria [34]. Age at diagnosis and disease phenotype (location and behaviour for CD, disease extent for UC) were classified according to the Montreal classification [35]. Phenotypic data were collected by patient questionnaire, interview and casenote review and were composed of demographics, dates of onset and diagnosis, disease location/extent, disease behaviour, surgical operations, smoking history and family history. Controls (n = 21) for these experiments were patients undergoing endoscopy for investigation of conditions not related to IBD, but who were otherwise healthy, e.g. surveillance colonoscopy for family history of colon cancer, or previous polyps. These subjects also gave a blood sample from which DNA was extracted and were included in the genetic analysis. All patients and controls gave informed, written consent. The demographics and disease characterisation of the patients and controls are shown in table 1.

**Table 1:** Patient demographics, disease characteristics and NOD2 genotype.

Demographic parameter	Subject Group		
	CD	UC	Controls
	N = 22	N = 26	N = 21
**Sex (M∶F)**	5∶17	14∶12	8∶13
**Age (years):** Median (range)			
At diagnosis	34.6 (10.4–71.6)	(10.3–79.7)	NA
At sampling	45.6 (25.9–74.6)	46.9 (18.9–80.9)	54.0 (22.2–80.6)
Disease duration	9.75 (0.18–35.1)	2.07 (0.03–27.4)	NA
**Positive Family History of IBD N (%)**	4 (18.2)	7 (28.0)	0 (0)
**Disease location/extent** * **N (%)**			
**CD Location**		NA	NA
Ileal disease (L1)†	7 (31.8)		
Colonic disease (L2)	11 (50.0)		
Ileo colonic (L3)	3 (13.6)		
Unknown	1 ( 4.6)		
**CD Behaviour**		NA	NA
Inflammatory	12 (54..5)		
Stricturing	7 (31.8)		
Penetrating	2 ( 9.1)		
Any penetrating perianal	4 (18.2)		
Unknown	1 ( 4.6)		
**UC Disease Extent**	NA		NA
Rectal disease (E1)		2 ( 7.7)	
Left-sided disease (E2)		11 (42.3)	
Extensive disease (E3)		11 (42.3)	
Unknown		2 ( 7.7)	
**Surgery prior to time of biopsy** ‡ **N (%)**			NA
Yes	10 (45.4)	2 ( 7.7)	
No	11 (50.0)	23 (88.5)	
Unknown	1 ( 4.6)	1 ( 3.8)	
**Disease activity** ‡‡ **N (%)**			NA
Active/inflamed	7 (31.8)	13 (50.0)	
Mildly inflamed	2 ( 9.1)	6 (23.1)	
Inactive/uninflamed	13 (59.1)	7 (26.9)	
**Smoking habit at Sampling**			
Current	8 (36.4)	5 (19.2)	4 (19.0)
Non	5 (22.7)	12 (46.2)	4 (19.0)
Ex	9 (40.9)	9 (34.6)	5 (23.8)
Unknown	0	0	8 (38.2)
**NOD2 mutation carriage** †† **(N %)**			
Any of R702W, G908R, 1007fs	4 (18.2)	2 ( 7.7)	2 ( 9.5)
None	16 (72.7)	22 (84.6)	16 (76.2)
Unknown	2 (9.1)	2 ( 7.7)	3 (14.3)

* Disease location/extent defined according to the Montreal Classification[35].

† One patient had upper GI disease with L1.

‡ Surgery was resection for CD, hemi-colectomy for UC.

‡‡ Disease activity as reported in pathology report.

†† NOD2 genotyping was carried out as previously described[36]. NA – not applicable.

Biopsies were obtained from the sigmoid colon. DNA samples obtained from blood [36] were available for 61 of these patients (20 CD, 24 UC and 17 HC).

### Organ culture method

Colonic pinch biopsies obtained at endoscopy were placed with luminal surface face up, on sterile metal grids over a central well of an organ culture dish (Falcon, UK), with the biopsies were at the liquid-air interface. The central well contained 2 ml Waymouth's medium (Gibco, UK) containing 100 U/ml penicillin (Sigma, UK), 100 µg/ml streptomycin (Sigma, UK), 50 µg/ml Gentamicin (Sigma, UK), 10% foetal calf serum (Gibco, UK), 300 µg/ml Ascorbic acid (Sigma, UK). Nicotine alone (1, 10, 100 µg/ml, Sigma, UK), LPS alone (1 µg/ml, from *Escherichia coli B4:111*), Sigma, UK) or nicotine and LPS together (100 and 1 µg/ml, respectively) were added to the medium in the dish and there was a medium only control. The doses of nicotine and LPS were those we have used previously [37]. Biopsies were incubated in a modular incubator chamber in a humidified atmosphere of 95% oxygen and 5% carbon dioxide under slight pressure. The time from collecting biopsies and placing them in culture was usually within 30 minutes. Biopsies were cultured for 24 hours, when the culture supernatants were collected and stored at −80°C for later analysis of HBD2 and cytokines (TNFα, IL1β, IL8 and IL10).

### HBD2 and cytokine analysis in supernatants

HBD2 was analysed in culture supernatants using a matched antibody pairs ELISA development kit (Peprotech, UK), according to manufacturer's instructions. TNFα, IL1β, IL8 and IL10 were also measured using matched antibody pair ELISA kits (Duosets, R&DSystems, UK), according to manufacturer's instructions.

### Sequencing of DEFB4 promoter region

The promoter region (∼1000 bp) of the *DEFB4* gene was investigated in 44 patient samples (14 CD, 16 UC and 14 HC). Potential NF-κB binding sites were identified in this region by the Transcription Element Search System (TESS) [38], which gave sites at −582 to −572 bp and −236 to −225 bp. Sites have also been described previously at positions −596 to −572 bp and −205 to −186 bp[39]. Primers spanning the region including all these sites were: *forward primers* AATCCCATCCTCCCATTCTC and TTTCACCAGCTCAGATCTCC; *reverse primers* GGAGATACAAGACCCTCATGG and CGAGAAGAGGAGATACAAGACCC. The PCR product was sequenced on an ABI Genetic Analyser at the MRC Human Genetics Unit, Edinburgh.

### Determination of DEFB4 copy number

DEFB4 genomic copy number was measured using established paralogue ratio testing (PRT) methods [40], [41] with modifications (Professor John Armour, personal communication) at the School of Biology, University of Nottingham.

### Statistical Analyses

Statistical tests were carried out using GraphPad Prism® version 4 (GraphPad Software, San Diego, CA, USA). Significance was defined at p<0.05. Comparisons of the microarray data used Kruskall-Wallis test with post-hoc Dunn's test for multiple comparisons or Mann Whitney tests. Comparison of unstimulated, LPS- and LPS+nicotine-stimulated HBD2 protein results between disease groups used Friedman tests or Wilcoxon signed rank tests. Comparisons across stimuli and/or disease groups were carried out using a two-way ANOVA. Correlations between cytokines and HBD2 production used a Spearman non-parametric correlation test.

## Results

### Expression of HBD2 mRNA throughout colon

**Figure 1:**
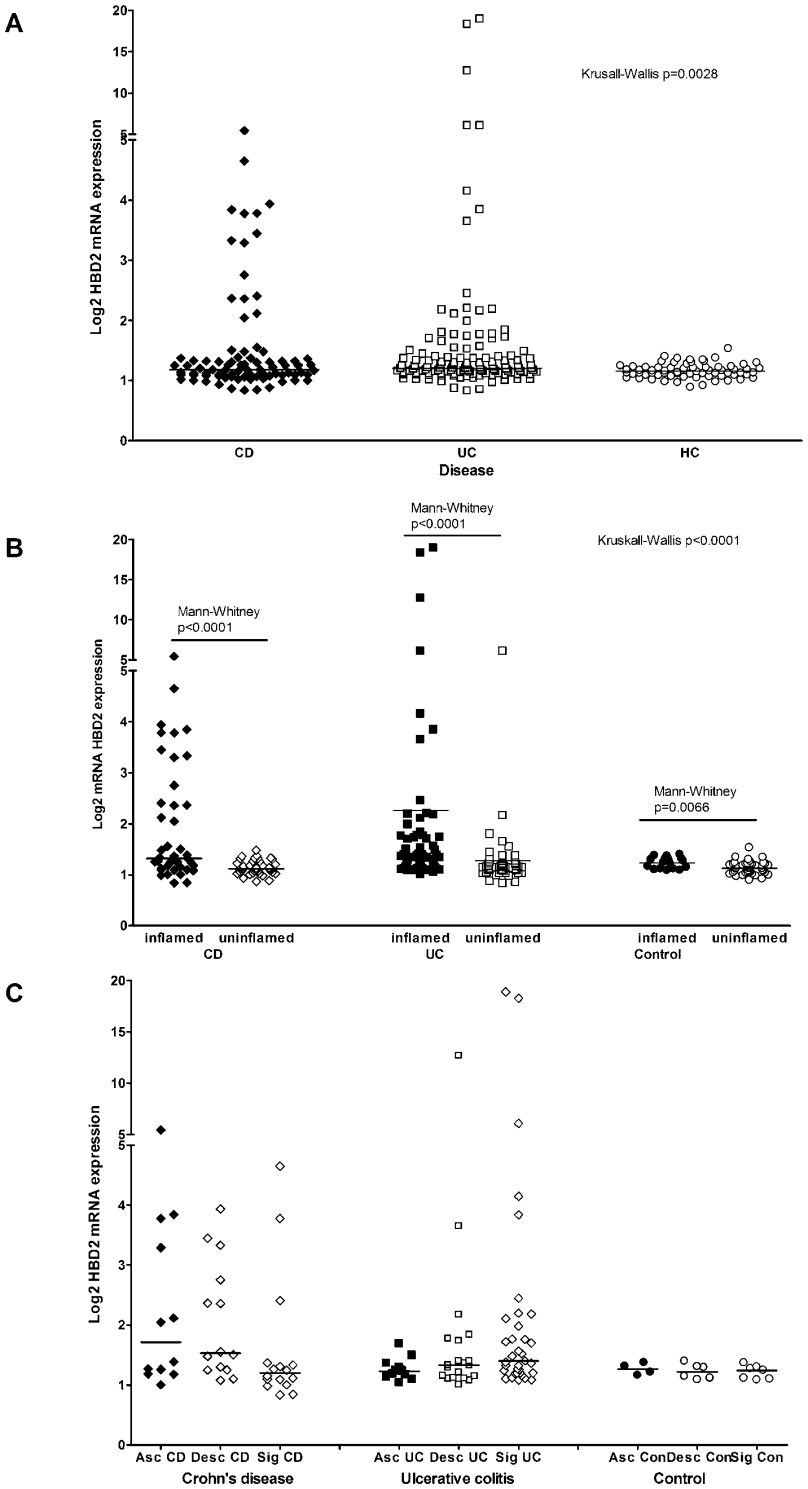
Expression of HBD2 mRNA with disease (A), inflammation (B) and colonic location of inflamed biopsies (C). All graphs show the log2 expression values from comparison with a reference marker (of value = 1) for HBD2 mRNA. Horizontal bars denote the median values for each analysis. P values from Mann-Whitney or Kruskall-Wallis tests are given, where significantly different.

To investigate HBD2 mRNA expression throughout the colon we mined the microarray data and found that the expression of HBD2 mRNA differed according to disease group (figure 1A), inflammation (figure 1B) and location in the colon (figure 1C). HBD2 expression is shown as the log2 mRNA expression relative to a control reference marker [23].

#### Effect of disease group

When biopsies from CD, UC patients and controls were compared, we found a significant difference (Kruskall-Wallis, p = 0.0028, figure 1A) driven by the differences between UC and controls (post-hoc Dunn's test, p<0.01). When CD biopsies were compared with controls, HBD2 mRNA was slightly but not quite significantly increased in CD patients (CD: mean = 1.550, median = 1.183, IQR 1.080–1.373 vs. controls mean = 1.157, median = 1.137, IQR 1.075–1.223, Mann-Whitney p = 0.0516). Comparison of UC and controls showed increased HBD2 expression in UC (UC mean = 1.808, median = 1.206, IQR 1.124–1.420, Mann-Whitney vs. controls p = 0.0004).

#### Effect of inflammation

When inflamed and uninflamed biopsies were compared, we found that for all three groups HBD2 mRNA expression was increased with inflammation (figure 1B; CD inflamed: mean = 1.952, median = 1.321, IQR 1.135–2.580 vs. CD uninflamed: mean = 1.138, median = 1.113, IQR 1.051–1.239, Mann-Whitney p<0.0001; UC inflamed: mean = 2.265, median = 1.330, IQR 1.171–1.761 vs. UC uninflamed: mean = 1.272, median = 1.156, IQR 1.076–1.257, Mann-Whitney p<0.0001; control inflamed: mean = 1.230, median = 1.247, IQR 1.116–1.313 vs. control uninflamed: mean = 1.131, median = 1.126, IQR 1.048–1.203, Mann-Whitney p = 0.0066). There were no differences when inflamed and uninflamed biopsies from the three groups were compared (Kruskall-Wallis inflamed p = 0.1215, uninflamed p = 0.2932).

#### Effect of colonic location

For inflamed biopsies from both CD and UC patients we found a differential gradient of HBD2 mRNA expression: in CD, the mean and median expression was highest in biopsies obtained from the ascending colon (mean = 2.316, median = 1.716, IQR 1.221–3.535), then descending colon (mean = 2.050, median = 1.530, IQR 1.251–3.042) with the lowest expression in sigmoid colon (mean = 1.593, median = 1.201, IQR 1.049–1.352); in UC the opposite pattern was seen with the lowest expression in the ascending colon (mean = 1.265, median = 1.234, IQR 1.049–1.328) then descending colon (mean = 2.043, median = 1.332, IQR 1.130–1.755) and highest expression in the sigmoid colon (mean = 2.735, median = 1.405, IQR 1.200–2.107). A gradient of expression was not seen in inflamed biopsies from control patients: ascending colon (mean = 1.267, median = 1.265, IQR 1.1890–1.345), descending colon (mean = 1.225, median = 1.220, IQR 1.103–1.354) and sigmoid colon (mean = 1.213, median = 1.247, IQR 1.100–1.301). HBD2 mRNA expression in inflamed sigmoid biopsies was higher in UC compared with controls (Mann-Whitney p = 0.0364).

### In organ culture, HBD2 protein was increased by LPS alone in CD and UC but not controls, and further increased by addition of nicotine in UC

**Figure 2:**
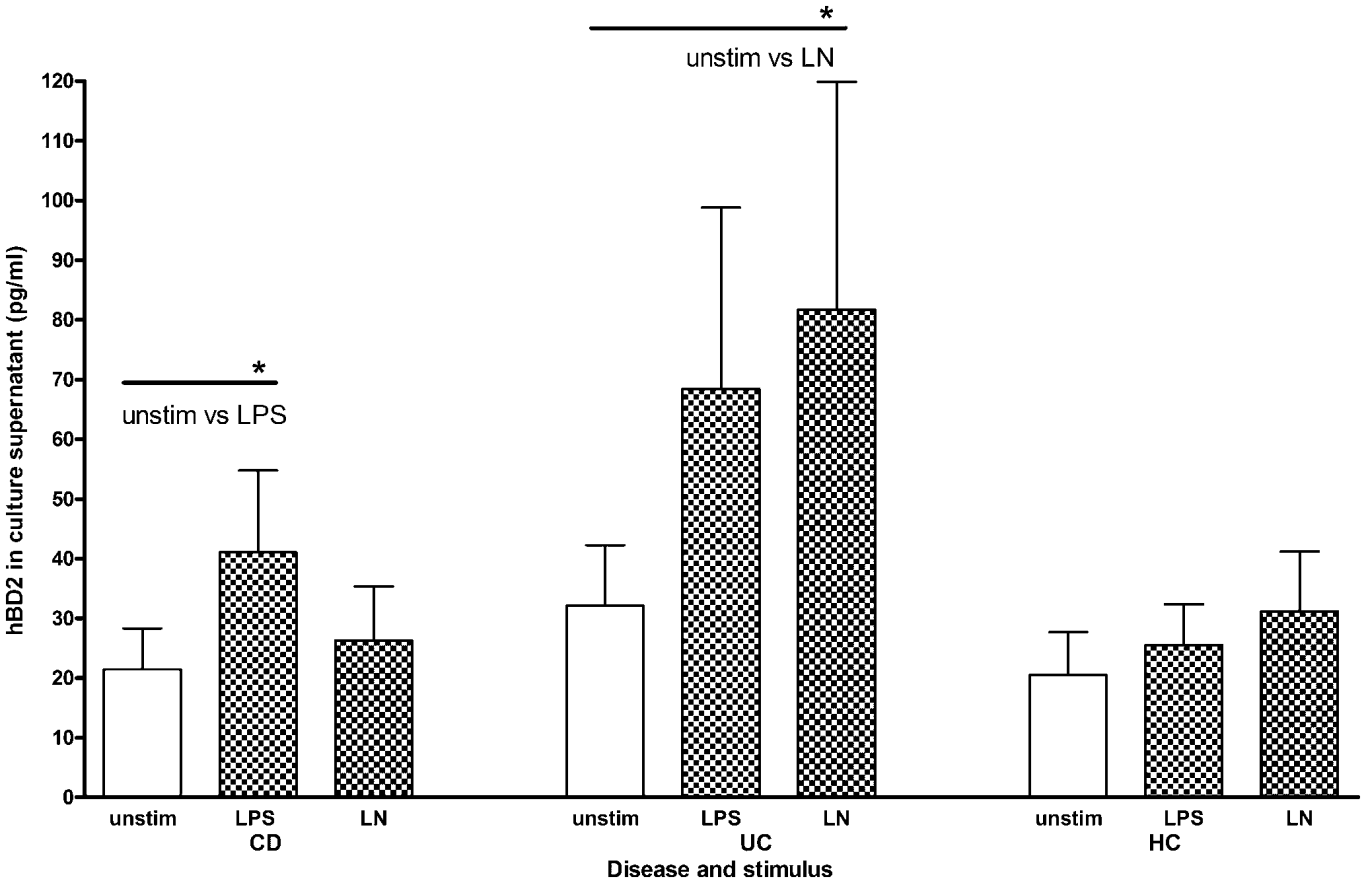
Production of HBD2 protein by LPS or LPS+nicotine in CD and UC patients and controls. HBD2 protein production (mean+sem) from culture supernatants of unstimulated or LPS- or LPS+nicotine (denoted LN)-stimulated biopsies in organ culture. * denotes P values of<0.05 from the results of Wilcoxon signed rank tests and the groups compared are shown. The actual p values are given in Results.

HBD2 production was measured in supernatants of unstimulated and stimulated biopsies from IBD patients and controls (figure 2). There was no difference in unstimulated HBD2 production between CD and UC patients and controls (CD unstimulated: mean = 21.45, median = 7.02, IQR 0–31.1 pg/ml; UC unstimulated: mean = 32.14, median = 8.892, IQR 0–43.0 pg/ml; control unstimulated: mean = 20.51, median = 8.64, IQR 0–37.1 pg/ml; Kruskall-Wallis p = 0.855).

In CD, LPS significantly induced HBD2 production from the biopsies (unstimulated: mean = 21.45, median = 7.02, IQR 0–31.1 pg/ml; LPS: mean = 41.07, median = 13.1, IQR 0.99–52.4 pg/ml Wilcoxon signed rank test p = 0.0375, figure 2). The increased production of HBD2 by LPS was not sustained by addition of nicotine (LPS+nicotine: mean = 26.34, median = 8.11, IQR 0–36.4 pg/ml, unstimulated vs. LPS+nicotine: Wilcoxon signed rank test p = 0.6872).

For UC, LPS increased production of HBD2 from biopsies but this was not significant in comparison to unstimulated biopsies (unstimulated: mean = 32.14, median = 8.892, IQR 0–43.0 pg/ml; LPS: mean = 68.41, median = 4.68, IQR 0–59.5 pg/ml, Wilcoxon signed rank test p = 0.2017). In UC, addition of nicotine to LPS further increased HBD2 production, which was significantly different from unstimulated biopsies (LPS+nicotine: mean = 81.73, median = 9.65, IQR 0–57.7 pg/ml, unstimulated vs. LPS+nicotine: Wilcoxon signed rank test, p = 0.0308, figure 2) and almost significantly different from stimulation with LPS alone (Wilcoxon signed rank test LPS vs LPS+nicotine p = 0.0786).

For controls there was no significant induction of HBD2 production on stimulation with LPS (unstimulated: mean = 20.51, median = 8.64, IQR 0–37.1 pg/ml; LPS: mean = 25.51, median = 16.2, IQR 0–35.0 pg/ml, Wilcoxon signed rank test p = 0.5436). There was no further significantly increased production of HBD2 on stimulation with LPS and nicotine (LPS+nicotine: mean = 31.15, median = 14.45, IQR 0–32.5 pg/ml, unstimulated vs LPS+nicotine Wilcoxon signed rank test p = 0.5784, figure 2).

There was no difference in HBD2 production when biopsies were stimulated with nicotine alone in CD or UC patients or controls.

### HBD2 production was associated with inflammation in UC but not CD

**Figure 3:**
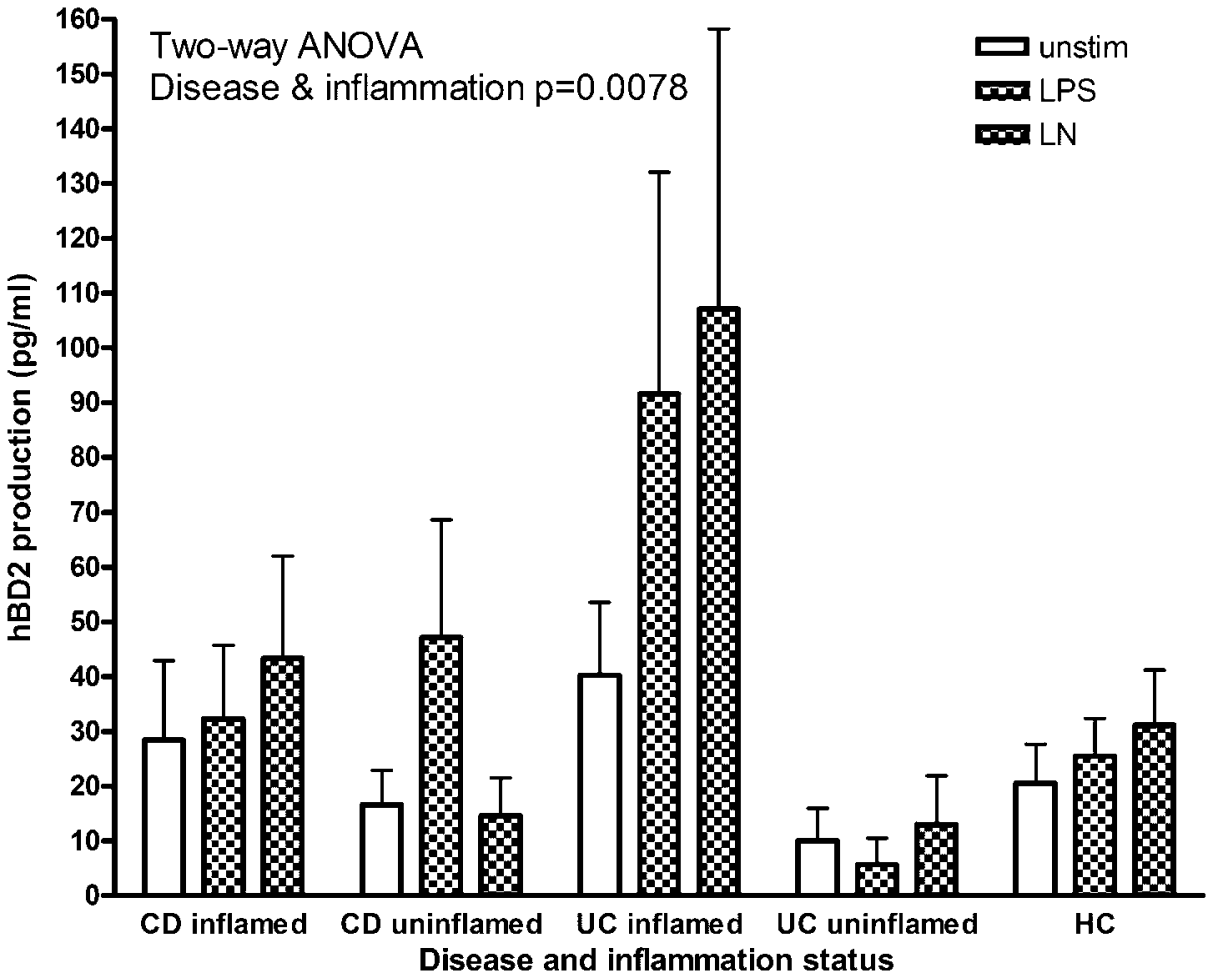
HBD2 production according to disease and inflammation status. HBD2 production (mean+sem) from biopsies from CD, UC patients or controls, unstimulated or stimulated with LPS or LPS+nicotine (denoted LN) and grouped according to disease and inflammation. P value given is from a two-way ANOVA compared by disease/inflammation or stimulation.

Comparison of HBD2 production by disease and inflammation (figure 3) showed that disease and inflammation significantly affected the levels of HBD2 produced (2-way ANOVA: disease/disease activity p = 0.0078), with less from the uninflamed biopsies of UC patients and the most from inflamed biopsies of UC patients. In CD, there was no difference in the amount of HBD2 produced with inflammation, but the pattern of HBD2 production differed: in inflamed biopsies HBD2 production in response to LPS+nicotine was increased, similar to that seen in UC, whereas in uninflamed biopsies it was reduced as previously noted when all biopsies from CD patients were analysed together.

### HBD2 increased with degree of inflammation in UC

**Figure 4:**
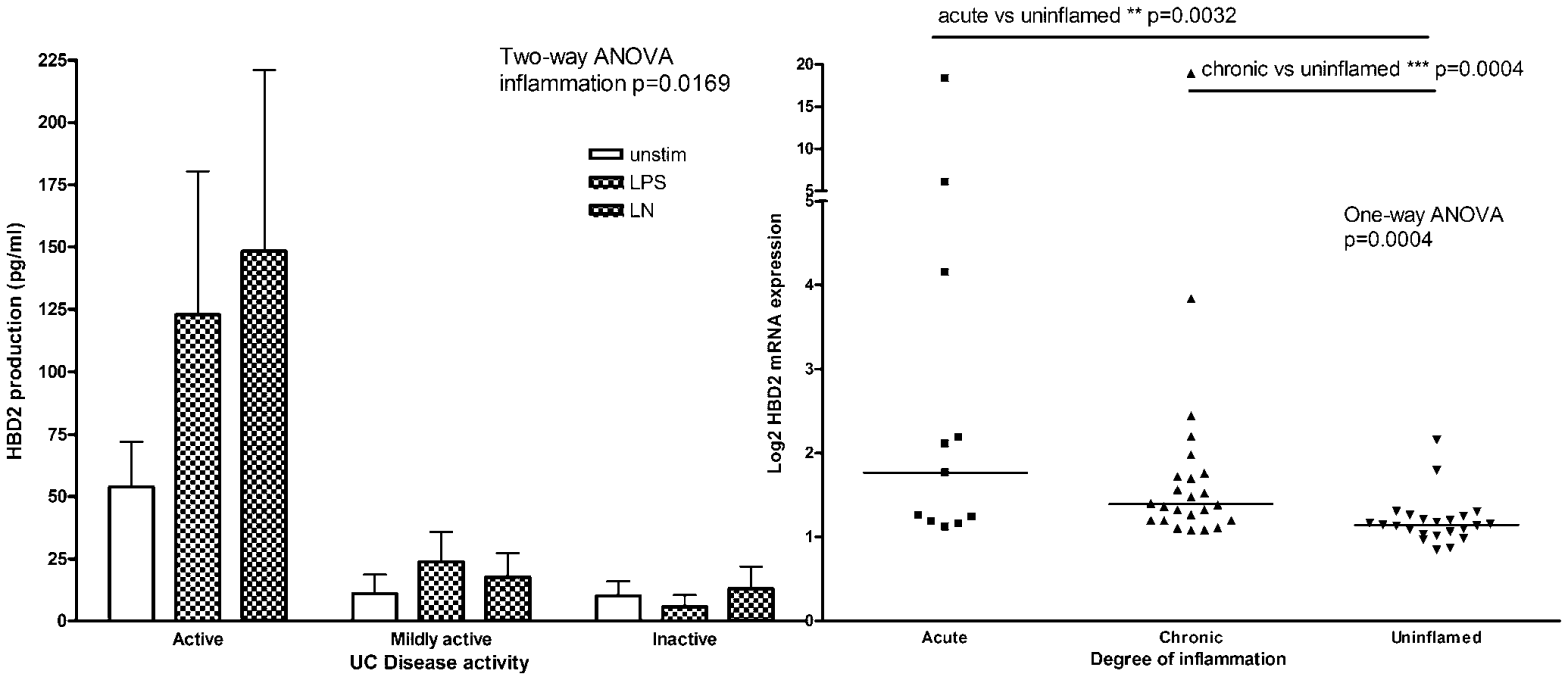
Increased HBD2 with inflammation in UC sigmoid colon is seen in protein (A) and mRNA (B). (A) HBD2 protein production from UC patients according to stimulation and inflammation status. Legend is as given for figure 3. The P value from a two-way ANOVA is given. (B) HBD2 mRNA expression for UC biopsies in sigmoid colon, grouped according to inflammation status[23]. Horizontal bars denote median values. P values are from Mann-Whitney tests and the groups compared are indicated. The P value from a one-way ANOVA is also given.

Further analysis of the HBD2 production in the sigmoid colon of UC patients showed that HBD2 increased with degree of inflammation; when disease activity of the samples was taken into account for the protein production, inflammation status was significantly different (p = 0.0169, two-way ANOVA, figure 4A). Similarly, from the microarray data higher HBD2 mRNA expression was shown in acutely inflamed biopsies (acute: median = 1.76, IQR 1.19–4.15) and chronically inflamed biopsies (chronic: median = 1.39, IQR 1.20–1.74) compared with uninflamed biopsies (uninflamed median = 1.14, IQR 1.03–1.25; acute vs. uninflamed Mann-Whitney p = 0.0032; chronic vs. uninflamed Mann-Whitney p = 0.0004, one-way ANOVA p = 0.004 figure 4B).

### Production of HBD2 differentially correlated with production of cytokines

The results of correlations between HBD2 production and cytokine production are presented in table 2. HBD2 positively correlated with IL8 production in UC patients and HC but not in CD patients. HBD2 production negatively correlated with IL1β in uninflamed, unstimulated biopsies from CD patients (p = 0.0396), but this was not seen in any other group.

**Table 2:** Correlations between HBD2 and IL1β, IL8 & IL10 production from unstimulated, LPS- and LPS+nicotine-stimulated biopsies obtained from patients and controls.

Biopsies	Correlation with IL1β			Correlation with IL8			Correlation with IL10		
Disease, stimulation and inflammation	Spearman r	95% CI	p value	Spearman r	95% C I	p value	Spearman r	95% CI	p value
**CD: unstimulated**									
All	0.0155	−0.420–0.455	0.945	−0.0885	−0.586–0.457	0.754	**0.429**	**−0.004–0.727**	**0.0463**
Inflamed	0.470	exact	0.213	0.0985	Exact	0.840	0.608	exact	0.0863
Uninflamed	**−0.575**	**−0.860– −0.0173**	**0.0396**	−0.347	Exact	0.389	0.298	−0.319–0.738	0.322
**CD: LPS-stimulated**									
All	−0.115	−0.552–0.335	0.611	−0.179	−0.643–0.381	0.523	**0.521**	**0.114–0.778**	**0.0129**
Inflamed	0.259	exact	0.493	0.216	Exact	0.662	**0.783**	Exact	**0.0172**
Uninflamed	−0.357	−0.766–0.259	0.231	−0.310	Exact	0.462	0.371	−0.243–0.773	0.212
**CD: LPS+nicotine stimulated**									
All	0.178	−0.276–0.567	0.428	0.171	−0.389–0.638	0.542	0.274	−0.180–0.632	0.217
Inflamed	0.377	exact	0.313	0.0721	Exact	0.906	**0.851**	**exact**	**0.0061**
Uninflamed	−0.0664	−0.607–0.517	0.829	0.317	Exact	0.428	−0.0724	−0.611–0.512	0.814
**UC: unstimulated**									
All	0.291	−0.121–0.617	0.149	**0.690**	**0.281–0.887**	**0.0031**	0.261	−0.153–0.597	0.198
Inflamed	0.374	−0.111–0.715	0.115	**0.890**	**0.634–0.970**	**0.0001**	0.283	−0.211–0.661	0.241
Uninflamed	−0.0935	exact	0.840	0.738	exact	0.333	0.115	exact	0.840
**UC: LPS-stimulated**									
All	0.130	−0.282– 0.502	0.527	**0.771**	**0.432–0.919**	**0.0005**	**0.456**	**0.0712–0.723**	**0.0192**
Inflamed	0.071	−0.409–0.519	0.774	**0.947**	**0.810–0.986**	**<0.0001**	0.434	−0.0403–0.748	0.0637
Uninflamed	0.0591	exact	0.906	0.775	exact	0.333	−0.307	exact	0.498
**UC: LPS+nicotine stimulated**									
All	0.234	−0.181–0.578	0.251	**0.808**	**0.531–0.937**	**0.0001**	**0.421**	**0.0285–0.701**	**0.0320**
Inflamed	0.313	−0.179–0.680	0.192	**0.873**	**0.588–0.965**	**0.0002**	**0.489**	**0.0300–0.778**	**0.0337**
Uninflamed	−0.0371	exact	0.964	0.949	Exact	0.0833	−0.231	exact	0.595
**HC: unstimulated**	0.0561	−0.397–0.487	0.809	0.229	−0.238–0.610	0.319	**0.516**	**0.0948–0.781**	**0.0167**
**HC: LPS-stimulated**	−0.0589	−0.489–0.394	0.799	**0.607**	**0.224–0.827**	**0.0035**	0.294-	−0.171–0.652	0.195
**HC: LPS+nicotine stimulated**	0.337	−0.125–0.678	0.136	**0.503**	**0.0771–0.773**	**0.0202**	0.266	−0.201–0.634	0.244

HBD2 production positively correlated with IL10 production in unstimulated and LPS-stimulated biopsies from CD patients but these correlations were lost when inflammation status was taken into account. Similarly HBD2 production from all LPS-stimulated biopsies from UC patients also correlated with IL10 production, but this did not hold when inflammation status was taken into account. For HC, HBD2 production from unstimulated biopsies correlated with IL10 but not for LPS-stimulated biopsies. There was no correlation between production of HBD2 and production of TNFα in any biopsies.

### HBD2 production was not associated with disease phenotype in CD or UC

There was no association in HBD2 production according to location (two-way ANOVA p = 0.376) or disease behaviour (two-way ANOVA, p = 0.813) in CD patients. In UC, an association with disease extent (two-way ANOVA p = 0.0042) was explained by the increased inflammation in the patients with most extensive disease. For both CD and UC there was no association with need for surgery (two-way ANOVA, p = 0.232, p = 0.781, respectively). There was no difference in HBD2 production according to smoking habit at time of sampling in any disease group (two way ANOVA for smoking habit, p = 0.760).

### No correlation between polymorphisms in the NF-κB binding sites and hBD2 production

HBD2 production has been shown to be abrogated by deletion of NF-κB binding sites in the HBD2 promoter [28]. To investigate further potential explanations for the increased HBD2 production in response to LPS in CD and UC but not HC, we sequenced the promoter region of the *DEFB4* gene, to ask whether IBD patients had polymorphisms at potential NF-κB binding sites, as identified by Transcriptional Element Search Software (TESS) [38]. NF-κB recognises the consensus sequence GGG*RN*T*YY*CC [42]. For the region between −596 bp to −572 bp, all patient sequences were found to be the same as that found in Ensembl. For the regions −236 to −225 bp and −205 to −186 bp, variations in sequence were found, but there was no polymorphism that changed the site into the NF-κB binding site consensus sequence.

There was no significant difference in hBD2 production from CD patients with sequence variations at any NF-κB binding sites (variants) than in those wild-type (WT) sequence (CD WT unstimulated mean = 29.43, median = 0, IQR 0–58.86; CD WT LPS mean = 12.62, median = 6.611, IQR 0–25.25; CD variants unstimulated mean = 20.55, median = 15.07, IQR 1.408–31.13; CD variants LPS mean = 55.14, median = 35.15, IQR 4.750–92.34, one-way ANOVA p = 0.2493). In UC patients, any differences in HBD2 production in the patients with WT sequence compared with those with variant sequence, were not significant (UC WT unstimulated mean = 30.53, median = 7.030, IQR 0–66.17; UC WT LPS mean = 91.94, median = 7.415, IQR 0–106.7; UC variants unstimulated mean = 7.304, median = 5.624, IQR 0–16.29; UC variants LPS mean = 2.516, median = 1.240, IQR 0–6.307, one-way ANOVA p = 0.5867). In HC patients, there were no differences in HBD2 production between unstimulated and stimulated biopsies from patients with variants or wild-type sequence (HC WT unstimulated mean = 20.40, median = 0, IQR 0–11.26; HC WT LPS mean = 21.74, median = 13.13, IQR 0–29.13; HC variants unstimulated mean = 17.16, median = 12.2, IQR 0–39.27; HC variants LPS mean = 18.04, median = 16.53, IQR 1.318–36.28, one-way ANOVA p = 0.4956).

### No difference in HBD2 production according to DEFB4 copy number

**Figure 5:**
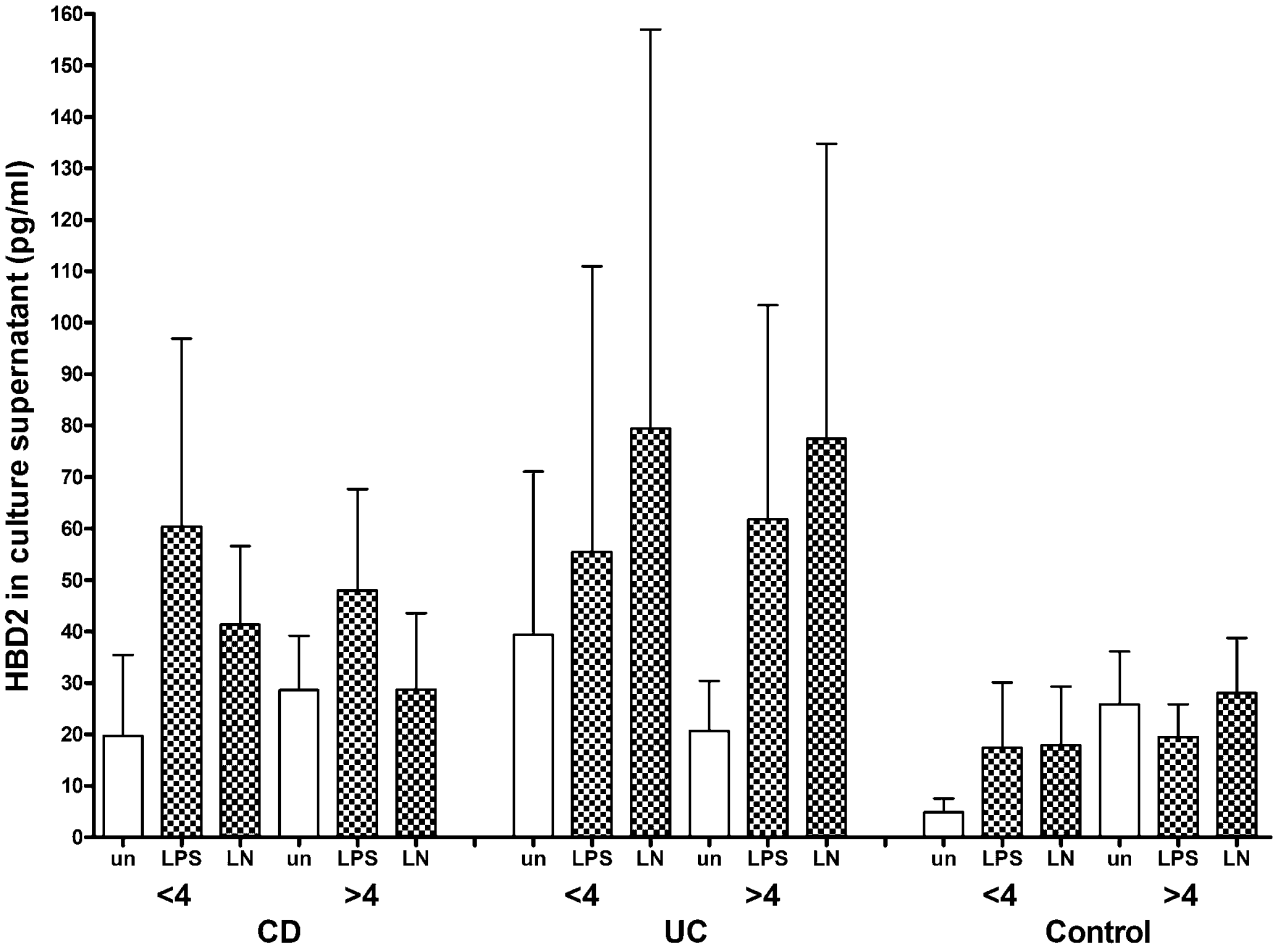
HBD2 production according to DEFB4 CNV. HBD2 production (mean+sem) from biopsies from CD, UC patients or controls, unstimulated or stimulated with LPS or LPS+nicotine (denoted LN) and grouped according to copy number.

HBD2 protein production (unstimulated, LPS- and LPS+nicotine-stimulated) from biopsies was compared by *DEFB4* gene copy number in patients and controls. As the median copy number found in our patients was 4, biopsies were classed as<4 or ≥4 and results are shown in figure 5. No differences were found with CNV and HBD2 production from unstimulated or stimulated biopsies in any patient group (CD: Kruskall-Wallis p = 0.4533, UC: Kruskall-Wallis p = 0.9413, HC Kruskall-Wallis p = 0.9525). When biopsies from inflamed and uninflamed tissue were each compared, the previously seen differential pattern of response to LPS+nicotine in inflamed and uninflamed CD biopsies was confirmed in this analysis but no differences were seen (Kruskall-Wallis p = 0.5348). Similarly in UC, while increased HBD2 protein was seen in inflamed biopsies, there were no differences according to copy number (Kruskall-Wallis p = 0.3905).

There was no significant correlation between copy number and HBD2 production, even when inflammation was taken into account.

## Discussion

The interest in anti-bacterial peptides as an innate mechanism of defence against bacteria has increased in recent years. In CD, this interest has been intensified by the association between NOD2 mutations and decreased αdefensin production from Paneth cells [20]–[22], while α-defensins have also been found to be dysregulated in UC [23]. HBD2 has been shown to be induced in the gut [27], [28] and upregulated in IBD [25], [26]. More recently, HBD2 has been shown to be induced by IL12, IL23, and IL27 in keratinocytes [43] and by IL17 in airway epithelium [44]. These processes may also have relevance to IBD, as the IL17/IL23 pathway has been shown to be differentially regulated in CD and UC [45].

The principal aims of the present study were to assess colonic production of HBD2 protein, to compare these data with studies of mRNA expression [25], [26], and to assess the factors which may be involved in the dysregulated responses observed. We have shown that HBD2 mRNA expression was differentially increased with inflammation in specific colonic locations in CD and UC, and went on to show that HBD2 protein production was increased by LPS but differentially modulated by addition of nicotine in CD and UC. These latter findings may have direct relevance to the clinical observation of the disparate effect of smoking in these diseases [7], [8], [11].

In previous studies, Wehkamp *et al* [25] suggested that HBD2 mRNA was higher in UC than CD and induced with inflammation in UC but not CD. However, in their study biopsies were obtained at random from different areas of the colon, which confound interpretation as our data showed HBD2 mRNA expression differed according to location within the inflamed colon. In the sigmoid colon we confirmed that HBD2 mRNA expression is indeed higher in UC than CD, and our results also demonstrate the same pattern of dysregulation at protein level. These observations concur with other studies that have shown that colonic expression of antimicrobial peptides is increased in UC compared with CD [22], [46]. The differential colonic expression in UC paralleled that of other genes involved in innate immunity also seen in this microarray dataset, especially the alpha defensins, matrix metalloproteins 3 and 7, IL8, CCL20 and TLR4 [23].

We have gone on to address the issue as to whether the dysregulated responses seen in IBD represent secondary, appropriate responses to disease processes or are part of the pathogenic mechanisms. The increased expression of HDB2 in inflamed tissue from UC patients was significantly higher than that seen in controls. HBD2 was increased by LPS in IBD patients and controls, confirming that HBD2 production is stimulated by bacterial products [46]–[48]. As HBD2 production is reduced with deletion of NF-κB binding sites in the promoter region of the HBD2 gene [28], we investigated whether there were more genetic polymorphisms at these sites in IBD patients than controls, but found no differences. The augmented responses in IBD patients require further investigation as to whether these are a direct response to the local bacterial flora, as suggested by a recent study that showed increased HBD2 mRNA in response to Staphylococcus enterotoxin B in cultured duodenal explants [49], and/or inflammation, or a resultant artefact of the dyregulated immunity as part of IBD disease pathogenesis [43]–[45].

Our data add to the growing body of evidence in favour of dysregulation of defensin production in IBD. We confirmed that the production of HBD2 is increased with inflammation in UC but not CD [25], [26] and correlated with IL8 production. Wehkamp *et al* found that HBD2 correlated with IL8 in inflamed but not uninflamed biopsies from CD patients [25] but this was not confirmed in our data. IL8 is a chemokine produced by epithelial cells which is chemotactic for neutrophils and has been shown to be increased in inflamed colonic tissue from UC patients [23]. The correlation between levels of HBD2 and IL8 in UC and HC was found when all biopsies were analysed together and remained significant when LPS-stimulation was considered alone, suggesting that HBD2 production in response to bacterial products might lead to neutrophil accumulation as part of a wider inflammatory response. IL8 and HBD2 production also correlated in unstimulated biopsies from UC but not HC, suggesting that in UC patients there may be some priming to bacterial motifs that would produce an exaggerated defensin response [50].

We investigated whether smoking habit at sampling or stimulation with nicotine alone had any effect on HBD2 production, but found no differences. However, an interesting differential effect was seen from co-stimulation with LPS and nicotine: LPS-induced HBD2 was maintained on addition of nicotine in inflamed and uninflamed UC as well as inflamed CD tissue but reduced by nicotine in uninflamed CD tissue. We appreciate that the concentrations of nicotine used in our study were higher than those found in blood or tissues [51]–[53]. Nicotine is thought to have anti-inflammatory effects [54] but may also be involved in promoting chronic inflammation [55]. A combination of pre-existent inflammation and LPS stimulation may reduce the anti-inflammatory nature of nicotine, though this might also depend on the nature of both the inflammation and the time of exposure to nicotine, as seen in animal models of the effects of nicotine on systemic inflammation [56]. Evidence from the respiratory literature has suggested that the function of a specific defensin, HNP1, was modified in smokers but not non-smokers leading to specific changes in its antibacterial properties [57], [58]. While further investigation is required to confirm and strengthen these data, it could be suggested that nicotine may affect the production and/or function of HBD2.

Surprisingly, our results showed that production of HBD2 protein from patient biopsies in *ex vivo* culture was not associated with the genomic copy number in CD, UC or controls. This is in some apparent contrast with results of previous studies of mRNA, which found direct correlations between copy number and amount of mRNA in mucosal biopsies from IBD patients [33] and in lymphoblastoid cell lines [29]. These differences could be due to post-transcriptional or -translational modifications of HBD2, or relate to the fact that HBD2 is an inducible gene rather than constitutively expressed [25], [59]. In a study of induction of β-defensins in gingival keratinocytes [60] it was found that HBD2 was consistently induced by IL1β and TNFα, but that LPS and other cytokines might have an effect in different individuals. In our biopsy cultures a variety of cytokines are produced, which might in turn affect HBD2 protein production [43], [44], [60]. While a simple relationship between genomic copy number and protein production might be expected, a recent study in psoriasis showed that serum HBD2 production was a reflection of the disease activity in these patients and not the copy number [61]. Thus the relative contributions of inflammation (local and systemic), stimulus and copy number need to be teased out.

In conclusion, we have shown that HBD2 is differentially expressed in CD and UC in response to colonic location and inflammation. HBD2 production differed in CD and UC in response to stimuli representative of the environmental factors of bacterial stimulation and smoking products and may contribute to and/or reflect the dysregulated innate immunity and disease pathogenesis in IBD. We were unable to identify germline mutations in the HBD2 gene that might be involved in disease-associated dysregulation, but other epistatic or epigenetic factors may be important.
